# Plague – an infectious disease challenge far from resolved

**DOI:** 10.1016/j.mmr.2026.100078

**Published:** 2026-10-30

**Authors:** Hagen Frickmann, Dorothea Franziska Wiemer, Jonas Schmidt-Chanasit

**Affiliations:** Department of Microbiology and Hospital Hygiene, Bundeswehr Hospital Hamburg, 22049 Hamburg, Germany; Institute for Medical Microbiology, Virology and Hygiene, University Medicine Rostock, 18057 Rostock, Germany; Department of Internal Medicine, Bundeswehr Hospital Hamburg, 22049 Hamburg, Germany; Department of Arbovirology and Entomology, Bernhard Nocht Institute for Tropical Medicine, 20359 Hamburg, Germany; Faculty of Mathematics, Informatics and Natural Sciences, Universität Hamburg, 20146 Hamburg, Germany

**Keywords:** Plague, *Yersinia pestis*, Antimicrobial treatment, Post-exposure prophylaxis, Vaccine development, Biopreparedness

Recently, Sidhu *et al.* [1] deciphered a molecular mechanism of evolutionary adaptation of *Yersinia pestis* (*Y. pestis*), the causative agent of plague, to decreasing density and interconnection of rodent host populations during the three historically documented plague pandemics. It consists of an incomplete depletion of the plasmid-located virulence gene *pla*, which mediates the conversion of plasminogen to plasmin and thus the lysis of fibrin clots, facilitating bacterial dissemination from the infection site to regional lymph nodes. In rodent models, strains with reduced *pla* copy numbers caused attenuated bubonic, but no attenuation of pneumonic or septicemic disease, and the prolonged survival of their hosts allowed continued transmission even in sparse host populations [1].

Such adaptation to evolutionary pressure improves our understanding of the dynamics of environmental persistence of highly virulent zoonotic pathogens like *Y. pestis*, which leads to ongoing and often fatal infections in humans. Between 2019 and 2025, 10 countries reported 3847 suspected human plague cases and 423 deaths to the World Health Organization (WHO), corresponding to a case fatality ratio of 11% [2], [3]. Most cases occurred in the Democratic Republic of the Congo and Madagascar, and thus in settings in which limited resources for infection prevention and control facilitate flea-borne transmission [2], [3].

After flea-borne transmission of *Y. pestis* from rodent hosts to humans, bubonic plague results from bacterial dissemination to regional lymph nodes after a short incubation time of 1 to 7 d [2], [3]. Without adequate antibiotic treatment, bubonic plague can disseminate via the bloodstream, resulting in often fatal bacteremia and secondary organ manifestations including pneumonic plague [2], [3]. The latter is highly contagious via inhalation of bacteria-containing respiratory particles, requiring patient management under barrier nursing conditions [2], [3]. Other transmission routes include direct contact with infected animal tissues and, rarely, ingestion of contaminated food [2], [3].

Pneumonic and septicemic plague can progress rapidly; delays in effective antibiotic treatment of even a few hours may be fatal. Clinical findings alone cannot reliably distinguish plague from other causes of pneumonia, sepsis, or febrile lymphadenopathy [4]. Epidemiological information including residence in endemic areas, animal or flea exposure, and contact with suspected cases strengthens clinical suspicion. However, recognizable epidemiological links may initially be absent, particularly following an intentional release. Surveillance case definitions should therefore support classification without becoming prerequisites for testing or treatment. If case definitions fail as diagnostic tools, empiric treatment of suspected sepsis is likely to be initiated with beta-lactam antibiotics in accordance with recommendations such as those of the Surviving Sepsis Campaign [5], resulting in a high risk of treatment failure, since beta-lactams lack clinical efficacy against *Y. pestis*. In contrast, early suspicion or diagnosis may facilitate target-specific treatment.

For decades, recommendations relied on observational data only, with aminoglycosides regarded as first-line agents, doxycycline, fluoroquinolones, chloramphenicol, sulfadiazine, and trimethoprim-sulfamethoxazole as alternatives, and chloramphenicol as an option for meningeal infection [6]. This has changed; for example, in the randomized IMASOY trial including 220 patients with bubonic plague, 10 d of oral ciprofloxacin were non-inferior to 3 d of parenteral aminoglycoside followed by 7 d of ciprofloxacin (treatment failure 9.0% vs. 8.1%), supporting fully oral management of uncomplicated bubonic disease in remote endemic settings [7]. After transmission-relevant exposure, monitoring of contacts and post-exposure prophylaxis with ciprofloxacin or doxycycline are recommended [6]. However, multiple, mainly plasmid-mediated resistance mechanisms may limit the therapeutic options, and even against the first-line drug streptomycin, *rpsL* gene mutation-based resistance has been demonstrated [8], stressing the need for both antimicrobial resistance surveillance and immediate testing. Diagnostically, F1 antigen-based rapid tests can be applied at the bedside within minutes and are highly sensitive, but their limited specificity requires confirmation by PCR or culture [9]. Molecular assays are rapid and sensitive, yet usually presuppose clinical suspicion, whereas culture additionally provides isolates for susceptibility testing and typing, but requires laboratory facilities with an adequate biosafety level in order not to endanger personnel handling high quantities of viable bacteria [6], [9].

*Y. pestis* has been included in the WHO list of emerging and potentially pandemic pathogens, and the development of vaccines providing reliable immune protection against all clinical manifestations of plague is ongoing [10]. To date, no vaccine with established efficacy against pneumonic plague is licensed, although several candidates, among them adjuvanted recombinant F1/V subunit vaccines, have entered clinical evaluation [10].

Accordingly, several research priorities emerge for the future medical management of plague. Safe-to-apply, ideally hypothesis-free diagnostic solutions that detect plague even when the clinical presentation is non-specific would allow earlier targeted therapy. Systematic resistance surveillance in endemic areas would help clinicians to choose effective initial regimens, and the oral treatment option now supported by randomized evidence [7] should be evaluated for pneumonic and septicemic disease as well. Immunological research, as mentioned at the outset [1], and vaccine development remain essential to establish reliable preventive strategies.

## Ethics approval and consent to participate

Not applicable.

## Authors’ contributions

HF, DFW, and JSC jointly wrote and corrected the manuscript. A first manuscript draft, which was afterwards adapted and broadened, was provided by HF. All authors read and approved the final manuscript.

## Consent for publication

Not applicable.

## Competing interests

The authors declare that they have no competing interests.

## Funding

Not applicable.

## Data Availability

Not applicable.
